# Berberine alleviates biofilm-associated immune-inflammatory injury in *Staphylococcus aureus*-induced osteomyelitis: insights from network pharmacology and experimental validation

**DOI:** 10.3389/fimmu.2026.1878634

**Published:** 2026-07-13

**Authors:** Xuan Deng, Lin Zhang, Jinglin Li, Fuyin Yang, Chengrui Peng, Jiaze Peng, Yang Yu, Xianpeng Huang, Xuxu Yang, Lidan Yang

**Affiliations:** 1 Department of Orthopedics, Affiliated Hospital of Zunyi Medical University, Zunyi, China; 2 Department of Orthopedics Center, Renhuai People’s Hospital, Zunyi, China

**Keywords:** berberine, biofilm, HIF-1α, osteomyelitis, PTGS2, *Staphylococcus aureus*

## Abstract

**Background:**

*Staphylococcus aureus* (SA)-induced osteomyelitis (OM) is a common orthopedic infection characterized by biofilm formation and persistent inflammation. Berberine (BBR), a natural isoquinoline alkaloid, exhibits antibacterial and anti-inflammatory activities. However, its therapeutic potential in OM and underlying mechanisms remain unclear. This study investigated the effects of BBR against SA-induced OM and explored potential mechanisms through network pharmacology and validation.

**Methods:**

An *in vitro* SA biofilm model was established to assess the effects of BBR on bacterial survival and mature biofilm structure using colony-forming unit (CFU) counting, light microscopy, and confocal laser scanning microscopy (CLSM). A mouse model of SA-induced OM was established. A clindamycin (CLD)-treated group was included *in vivo* as a positive antibiotic comparator to benchmark the antibacterial efficacy of BBR and assess the limitations of this compound. Biofilm formation on implants was examined by scanning electron microscopy (SEM). Serum inflammatory mediators were measured by enzyme-linked immunosorbent assay (ELISA), and histopathological changes in peri-implant bone were evaluated by hematoxylin and eosin (H&E) staining. Network pharmacology and molecular docking were performed to identify targets and pathways, and key proteins were validated by Western blotting.

**Results:**

BBR significantly reduced bacterial viability within biofilms and decreased CFU counts *in vitro*. Microscopic observations showed disrupted biofilm architecture and reduced biofilm coverage after treatment. *In vivo*, compared with the untreated OM group, CLD produced a more pronounced reduction in bacterial load than BBR. BBR significantly alleviated bone destruction, reduced implant-associated biofilm formation, decreased inflammatory cell infiltration, and improved tissue morphology. ELISA results showed that BBR markedly reduced pro-inflammatory mediator levels. Network pharmacology identified prostaglandin-endoperoxide synthase 2 (PTGS2) as a key target and implicated the hypoxia-inducible factor 1 (HIF-1) signaling pathway. Molecular docking indicated favorable binding between BBR and PTGS2. Western blotting showed that BBR downregulated PTGS2 and HIF-1α expression in infected tissues.

**Conclusion:**

These findings suggest that BBR exerts protective effects against SA-induced OM through antibiofilm and anti-inflammatory activities. However, its antibacterial efficacy was weaker than that of CLD, indicating that BBR should not be regarded as a replacement for antibiotics but rather as an adjunctive therapy for biofilm-associated osteomyelitis.

## 1 Introduction

Osteomyelitis (OM) is a severe orthopedic infection, and *Staphylococcus aureus* (SA) is one of its most common causative pathogens (1, 2). Recent studies have shown that biofilm formation is a key mechanism underlying persistent SA infection and antibiotic tolerance. Bacteria within biofilms exhibit markedly enhanced tolerance to antimicrobial agents and host immune responses, making the infection difficult to eradicate completely (3–5). Consequently, SA-induced OM is characterized by an insidious onset, frequent recurrence, and prolonged treatment, which severely impair patients’ quality of life and pose major challenges to clinical management. In addition to persistent bacterial colonization, excessive or dysregulated host inflammatory responses play a critical role in OM progression (6). Following SA infection, the innate immune system is rapidly activated, triggering the release of pro-inflammatory cytokines and the activation of multiple inflammatory pathways, thereby continuously amplifying local inflammation (7). A persistent inflammatory microenvironment not only causes tissue edema, vasodilation, and cellular injury but also exacerbates bone destruction by promoting osteoclast activation and bone resorption (5). Therefore, therapeutic strategies for chronic bone infection should not only inhibit pathogen growth but also regulate abnormal inflammatory responses, thereby preventing sustained bone destruction and promoting tissue repair.

Berberine (BBR) is a natural isoquinoline alkaloid extracted from traditional Chinese medicines such as *Coptis chinensis* and *Phellodendron chinense*. It exhibits a wide range of pharmacological activities, including antibacterial, anti-inflammatory, antioxidant, and immunomodulatory effects (8–10). Previous studies have shown that BBR can interfere with SA biofilm biology through several mechanisms. For example, BBR has been reported to inhibit methicillin-resistant Staphylococcus aureus (MRSA) biofilm formation by affecting the aggregation of phenol-soluble modulins into amyloid fibrils, and its antibiofilm activity has also been evaluated in SA isolates associated with prosthetic joint infection. However, the effects of BBR may vary among bacterial sequence types, and subinhibitory concentrations may even enhance biofilm formation in some SA strains. In addition, BBR has been shown to act synergistically with other antimicrobial agents and to modulate the accessory gene regulator (Agr) system, thereby influencing biofilm formation or dispersal (11–14). BBR-based delivery strategies, including liposomal and aptamer/graphene oxide-based systems, have also been explored to improve antibiofilm efficacy against MRSA (15, 16). Nevertheless, most existing studies have focused on *in vitro* planktonic or biofilm models, isolates associated with prosthetic joint infection, or drug-combination and delivery systems. Systematic evidence regarding the role of BBR in SA-induced osteomyelitis, particularly its effects on implant-associated mature biofilms and biofilm-associated immunoinflammatory injury, remains limited. Moreover, the host-related molecular mechanisms underlying the protective effects of BBR in this context have not been fully elucidated.

With the development of systems biology, network pharmacology has emerged as a useful strategy for investigating the multicomponent, multitarget, and multipathway mechanisms of traditional Chinese medicines (17, 18). By integrating drug-target prediction, disease-associated gene screening, and molecular docking, this approach can systematically identify candidate therapeutic targets for subsequent experimental validation (19).

Accordingly, the present study aimed to systematically evaluate the antibacterial and antibiofilm effects of BBR and its anti-inflammatory activity using an *in vitro* SA biofilm model and a mouse model of OM. In parallel, network pharmacology and molecular docking were used to identify candidate targets, followed by preliminary experimental validation. This study sought to elucidate the protective effects and potential molecular mechanisms of BBR in SA-induced OM, thereby providing a preclinical basis for further investigation and identifying potential therapeutic targets.

## 2 Materials and methods

### 2.1 Main reagents and materials

Berberine (BBR; purity ≥98%) and clindamycin (CLD) were purchased from Solarbio (China). *Staphylococcus aureus* (*SA*; ATCC 25923) was kindly provided by the clinical laboratory of a tertiary hospital in China. Tryptic soy broth (TSB) and tryptic soy agar (TSA) were obtained from Solarbio (China). Enzyme-linked immunosorbent assay (ELISA) kits for interleukin-6 (IL-6), C-reactive protein (CRP), interleukin-1 beta (IL-1β), and interleukin-10 (IL-10) were purchased from Jiangsu Jingmei Biotechnology Co., Ltd. (China). Female C57BL/6J mice weighing 20 ± 2 g were obtained from Beijing SPF Biotechnology Co., Ltd. (China). Antibodies against prostaglandin-endoperoxide synthase 2 (PTGS2) and hypoxia-inducible factor 1 alpha (HIF-1α), together with other Western blot reagents, were purchased from Immunoway and Bioswamp (China).

### 2.2 *In vitro* bacterial experiments

SA was inoculated onto blood agar plates and cultured at 37°C for 24 h. A single colony was then transferred to TSB and cultured at 37°C for 24 h. A portion of the bacterial suspension was centrifuged at 1,000 rpm for 5 min; the supernatant was discarded, and the pellet was resuspended in 1 mL glycerol and stored at −80°C until use. Before each experiment, the bacterial suspension was adjusted to an optical density at 600 nm (OD600) of 0.6, corresponding to approximately 5 × 10^8 colony-forming units per milliliter (CFU/mL), as verified by serial dilution and plate counting. All *in vitro* experiments included three technical replicates per condition and were independently repeated three times.

#### 2.2.1 Determination of the minimum inhibitory concentration of BBR against SA

The minimum inhibitory concentration (MIC) of BBR against SA was determined using a 2,3,5-triphenyltetrazolium chloride (TTC)-assisted broth dilution method. Twofold serial dilutions were prepared in 96-well plates to yield final BBR concentrations ranging from 0.00625 to 3.2 mg/mL. The bacterial suspension was added to each well and incubated at 37°C for 24 h. TTC was then added, and color development was assessed. The lowest BBR concentration showing no color development was recorded as the MIC.

#### 2.2.2 Biofilm formation and determination of the minimum biofilm eradication concentration

Sterile 22 × 22 mm coverslips were placed in 6-well plates, and 4 mL of the prepared bacterial suspension was added to each well. The plates were incubated at 37°C for 1–72 h. After incubation, the coverslips were gently washed with phosphate-buffered saline (PBS) to remove planktonic bacteria, fixed with methanol for 15 min, stained with 0.1% crystal violet for 15 min, and rinsed with PBS. Biofilm formation was observed by light microscopy.

To determine the time point at which biofilm coverage reached a plateau, crystal violet-stained biofilms from different incubation times were photographed using identical microscope settings. For each time point, at least five randomly selected fields from each coverslip were analyzed using ImageJ. The crystal violet-positive area was quantified as an index of biofilm surface coverage. The time point with the greatest and subsequently stable crystal violet-positive area was considered to represent mature biofilm formation and was selected for subsequent analyses.

After mature biofilms had formed, they were treated with serial concentrations of BBR for 24 h in 96-well plates. The wells were then washed with PBS, fixed with methanol, incubated with 3-(4,5-dimethylthiazol-2-yl)-2,5-diphenyltetrazolium bromide (MTT), and the resulting product was solubilized in dimethyl sulfoxide (DMSO). Antibiofilm activity was evaluated based on color development, and the minimum biofilm eradication concentration (MBEC) was operationally determined. Each concentration was tested in triplicate wells, and the experiment was independently repeated three times.

#### 2.2.3 Effects of BBR on SA biofilms and viable bacteria within biofilms at different time points

The experimental groups were established as follows: the control group (Con), in which 2 mL of TSB was added to each well; and the BBR group, in which 2 mL of BBR solution at a concentration of 3.2 mg/mL was added to each well. After mature biofilms had formed in 6-well plates, they were exposed to BBR for 6, 12, or 24 h, whereas an equal volume of TSB was added to the corresponding control wells. After treatment, crystal violet staining was performed to assess changes in biofilm surface coverage rather than three-dimensional biofilm morphology. Biofilms were imaged using identical microscope settings, and at least five randomly selected fields per sample were analyzed using ImageJ. Biofilm coverage was quantified as the crystal violet-positive area.

To determine the number of viable bacteria within biofilms, the biofilms were washed with PBS and detached by ultrasonication at 35 kHz for 15 min, followed by vortexing for 5 min. The resulting bacterial suspension was serially diluted and plated on TSA. After incubation at 37°C for 24 h, colonies were counted, and CFU/mL values were calculated to assess bacterial burden. In addition, biofilms treated for 24 h were stained using a LIVE/DEAD kit (Sheng’er Biotechnology, China). Bacterial viability and distribution within the biofilms were examined by confocal laser scanning microscopy (CLSM), and fluorescence images were quantified using ImageJ.

### 2.3 Animal experiments

#### 2.3.1 Establishment and sample collection of the SA-induced OM mouse model

Forty 7-week-old female C57BL/6J mice were housed under specific pathogen-free (SPF) conditions at 22 ± 1°C, 50 ± 5% relative humidity, and a 12-h light/dark cycle. The mice were randomly allocated to four groups (n = 10 per group): the control group (Con), which received sterile steel pins; the osteomyelitis group (OM), which received bacteria-coated steel pins; the BBR group, which received bacteria-coated steel pins plus oral BBR; and the CLD group, which received bacteria-coated steel pins plus oral CLD.

For bacterial coating, sterile steel pins were immersed in an SA suspension adjusted to an OD600 of 0.6, corresponding to approximately 5 × 10^8 CFU/mL. The pins were incubated in the bacterial suspension at 37°C for 2 h to allow bacterial adhesion. Before implantation, the bacteria-coated pins were gently rinsed with sterile PBS to remove loosely attached planktonic bacteria. Sterile steel pins incubated in sterile TSB under the same conditions were used for the Con group.

Before surgery, mice were anesthetized by intraperitoneal injection of sodium pentobarbital at 40 mg/kg. An adequate depth of anesthesia was confirmed by the absence of a toe-pinch reflex. An incision was then made along the medial side of the right anterior tibial muscle, and a steel pin was implanted. SA-coated steel pins were implanted in the OM, BBR, and CLD groups, whereas sterile steel pins were implanted in the Con group. The incision was closed in layers after surgery.

Beginning on postoperative day 1, mice in the BBR and CLD groups received BBR at 100 mg/kg and CLD at 30 mg/kg, respectively, by oral gavage once daily for 21 consecutive days. Mice in the Con and OM groups received an equal volume of the corresponding vehicle according to the same schedule. Drug doses were adjusted according to body weight. Three weeks after model establishment, the mice were anesthetized by intraperitoneal injection of sodium pentobarbital at 40 mg/kg and then euthanized by cervical dislocation. Death was confirmed by the absence of heartbeat and respiratory movement. Blood samples, implants, and tibial tissues were collected for subsequent analyses.

All animal experiments were conducted in accordance with national ethical guidelines for animal research and were approved by the Ethics Committee of the Affiliated Hospital of Zunyi Medical University (approval number: zyfy-an-2023-0319).

#### 2.3.2 Analysis of bacterial load and scanning electron microscopy of biofilms on implant surfaces

The implants were removed and placed in PBS, followed by ultrasonication at 35 kHz for 15 min and vortexing for 5 min to detach bacteria from the implant surface. The suspension was serially diluted 10-fold (10−1 to 10−4), and 20 μL of each dilution was plated on TSA. After incubation at 37°C for 24 h, bacterial colonies were counted and expressed as CFU/mL.

For scanning electron microscopy (SEM), separate implants were used to preserve the surface biofilm structure. The implants were gently rinsed three times with PBS to remove tissue debris and loosely attached planktonic bacteria and were then immediately placed in Eppendorf tubes containing 1 mL of electron microscopy fixative. The samples were fixed for 2 h at room temperature and stored at 4°C until further processing. Before SEM preparation, the fixed implants were washed three times with 0.1 M PBS (pH 7.4) for 15 min each. The samples were then postfixed with 1% osmium tetroxide in 0.1 M PBS for 90 min at room temperature in the dark, followed by three washes with 0.1 M PBS for 15 min each. Subsequently, the samples were dehydrated through a graded ethanol series (30%, 50%, 70%, 80%, 90%, 95%, and 100%), with two 15-min incubations at each concentration. After dehydration, the samples were treated with isoamyl acetate for 15 min and dried using a critical-point drying apparatus. The dried samples were mounted on conductive carbon adhesive tape and sputter-coated with gold for 30 s using an ion sputter coater. Finally, biofilms on the implant surfaces were examined and imaged by SEM.

#### 2.3.3 Hematoxylin and eosin staining of peri-implant bone tissues

Tibial samples from the different experimental groups were fixed with 4% paraformaldehyde. The samples were decalcified in 10% ethylenediaminetetraacetic acid (EDTA) solution for 4 weeks, embedded in paraffin, and serially sectioned at a thickness of 4 μm. Hematoxylin and eosin (H&E) staining was performed according to the manufacturer’s instructions, and images were acquired using a light microscope.

#### 2.3.4 ELISA measurement of serum inflammatory mediators

Whole-blood samples were collected and allowed to clot overnight at 4°C. Serum was obtained by centrifugation at 1,000 × g for 20 min at 4°C. Standards and samples were added according to the ELISA kit instructions. After incubation with horseradish peroxidase (HRP)-conjugated antibodies and plate washing, tetramethylbenzidine (TMB) substrate was added for color development. The reaction was stopped by adding stop solution, and the optical density at 450 nm (OD450) was measured using a microplate reader. The concentrations of IL-6, IL-1β, IL-10, and CRP were calculated from the corresponding standard curves.

### 2.4 Network pharmacology

#### 2.4.1 Network pharmacology analysis

The two-dimensional (2D) and three-dimensional (3D) structures of BBR were obtained from the PubChem database (https://pubchem.ncbi.nlm.nih.gov/). The structural information was then submitted to SwissTargetPrediction (http://www.swisstargetprediction.ch/) to predict potential protein targets of BBR.

Gene-expression data related to OM were obtained from the GSE166522 dataset in the Gene Expression Omnibus (GEO) database. Differential expression between OM samples and matched control samples was analyzed using the DESeq2 package in R (20). Differentially expressed genes (DEGs) were selected using P < 0.05 and a dynamic threshold of |log2 fold change (log2FC)| > [mean(|log2FC|) + 2 standard deviations of |log2FC|]. Gene Ontology (GO) and Kyoto Encyclopedia of Genes and Genomes (KEGG) enrichment analyses of the identified DEGs were then performed using the clusterProfiler and enrichplot packages in R (21, 22).

Genes related to SA biofilms were retrieved from the GeneCards database (https://www.genecards.org/). Predicted BBR targets, OM-associated DEGs, and SA biofilm-related genes were intersected to identify candidate core regulatory genes.

Volcano plots were generated using the ggplot2 package in R, and heatmaps were generated using the pheatmap package to visualize DEG expression patterns (23).

#### 2.4.2 Molecular docking

The three-dimensional structures of candidate target proteins were obtained from the RCSB Protein Data Bank (PDB). Experimental structures with the highest available resolution were preferentially selected. If no suitable experimental structure was available, a model was generated from the protein sequence using SWISS-MODEL or AlphaFold and assessed for quality. Protein structures were prepared in PyMOL by removing water molecules, co-crystallized ligands, and other heteroatoms, followed by the addition of hydrogen atoms. The BBR structure was downloaded from PubChem, converted to PDB format with Open Babel, and protonated. AutoDockTools was used to prepare protein and ligand PDBQT files and assign docking parameters. Docking was performed with AutoDock Vina using a grid box encompassing the target binding site. Multiple poses were generated, and the pose with the lowest predicted docking score was selected. More negative Vina scores were interpreted as indicating more favorable predicted binding. Finally, PyMOL was used to visualize the protein-ligand complex and identify key interacting residues, thereby characterizing the predicted binding mode of BBR to PTGS2.

#### 2.4.3 Western blot analysis of key proteins

Total protein was extracted from tibial lesion or control tissues using radioimmunoprecipitation assay (RIPA) lysis buffer. Proteins were separated by sodium dodecyl sulfate-polyacrylamide gel electrophoresis (SDS-PAGE) and transferred to polyvinylidene fluoride (PVDF) membranes. After blocking with 5% nonfat milk, the membranes were incubated overnight at 4°C with primary antibodies against glyceraldehyde-3-phosphate dehydrogenase (GAPDH; Immunoway, YM8394), HIF-1α (Immunoway, YM8735), and PTGS2 (Bioswamp, PAB31107). The membranes were then incubated with the secondary antibody (Bioswamp, SAB48169) for 1 h at room temperature. Protein bands were visualized using a Western blot imaging system, and band intensities were quantified using ImageJ.

### 2.5 Statistical analysis

R version 4.4.0 was used for bioinformatics analyses and visualization. Experimental data were analyzed using SPSS version 29.0 (IBM, USA) and GraphPad Prism version 10.0 (GraphPad Software, USA). Normality was assessed using the Shapiro-Wilk test, and data are presented as the mean ± standard error of the mean. Comparisons between two groups were performed using an independent-samples *t test*. For comparisons among multiple groups, one-way analysis of variance (ANOVA) followed by Tukey’s *post hoc* test was used when the data were normally distributed and variances were homogeneous. Otherwise, the Kruskal-Wallis test followed by Dunn’s multiple-comparison test was used. All tests were two-tailed, and *P* < 0.05 was considered statistically significant.

## 3 Results

### 3.1 Determination of the MIC and MBEC of BBR against SA

**Figure 1:**
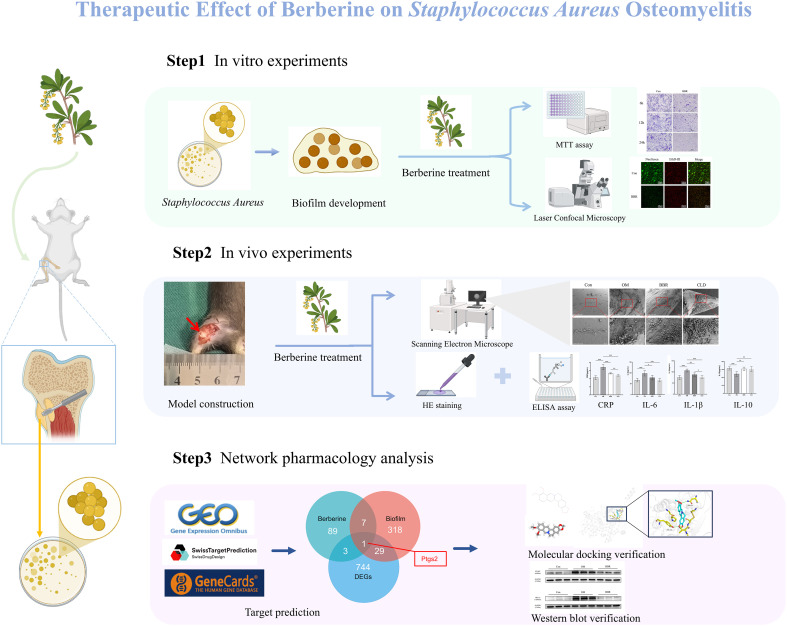
Flowchart of the overall experimental design.

**Figure 2:**
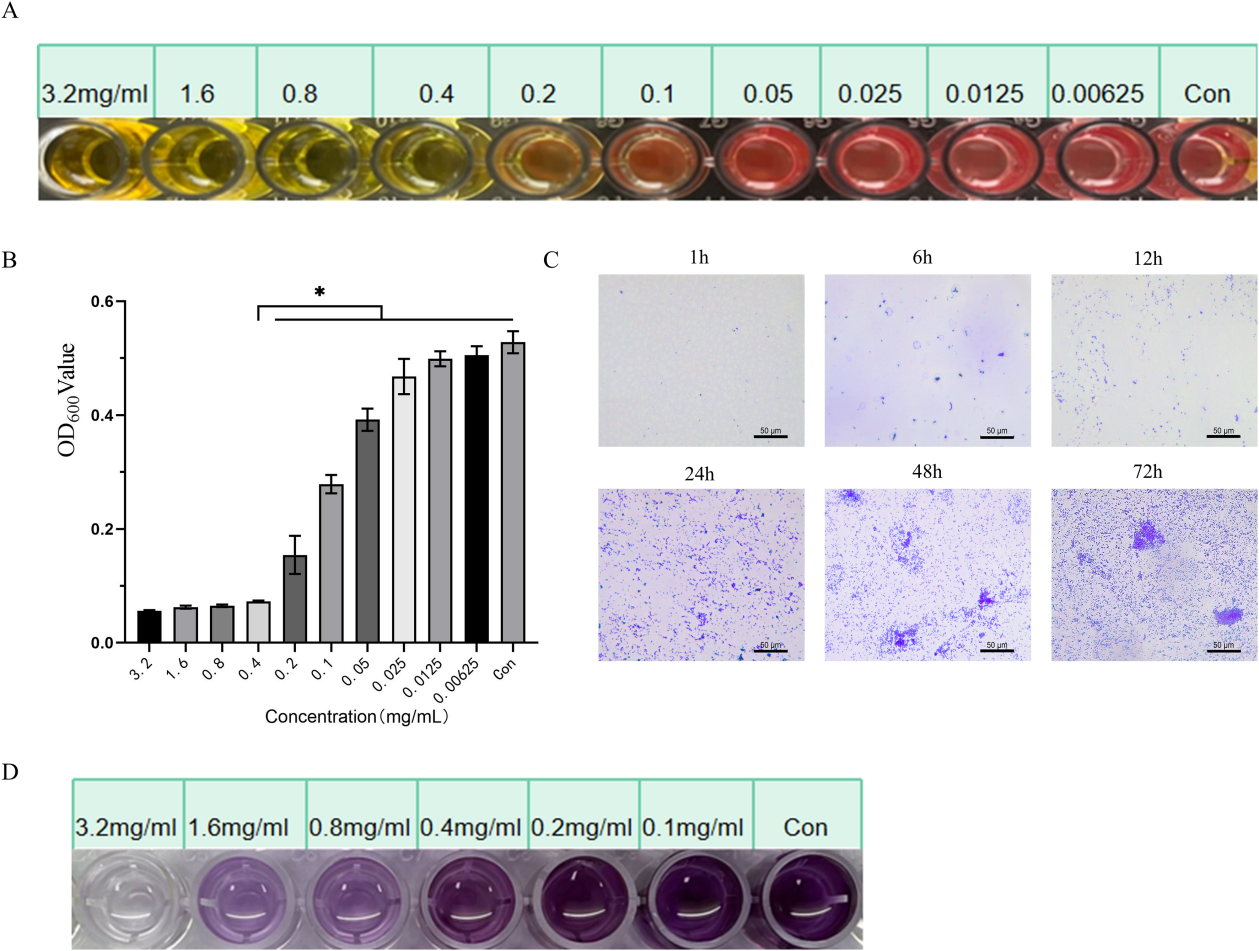
Effects of BBR on the growth, biofilm formation, and biofilm eradication of *S. aureus*. **(A)** TTC assay showing the effects of different concentrations of BBR on bacterial viability. **(B)** Quantitative analysis of the MIC. **(C)** Crystal violet staining of biofilms formed at different incubation times (1–72 h). Scale bar = 50 μm. **(D)** MTT assay of mature biofilms after treatment with different concentrations of BBR. *P < 0.05.

The experimental workflow is shown in **Figure 1**. The antibacterial activity of BBR against SA was evaluated using the TTC assay. The results showed that the MIC of BBR against SA was 0.4 mg/mL (**Figures 2A, B**). Crystal violet staining was used to monitor biofilm formation on coverslips. Biofilm coverage and staining intensity increased from 1 to 48 h, whereas the values at 48 and 72 h were comparable, indicating that biofilm formation had reached a relative plateau by 48 h (**Figure 2C**). Therefore, 48-h biofilms were used as mature biofilms in subsequent experiments. Mature biofilms formed in 96-well plates were treated with different BBR concentrations for 24 h and assessed using the MTT assay. The lowest tested concentration showing no detectable color development was 3.2 mg/mL and was operationally designated as the MBEC. Accordingly, 3.2 mg/mL BBR was used in subsequent *in vitro* experiments (**Figure 2D****).**

### 3.2 Effects of BBR on biofilms and viable bacteria within biofilms at different time points

**Figure 3:**
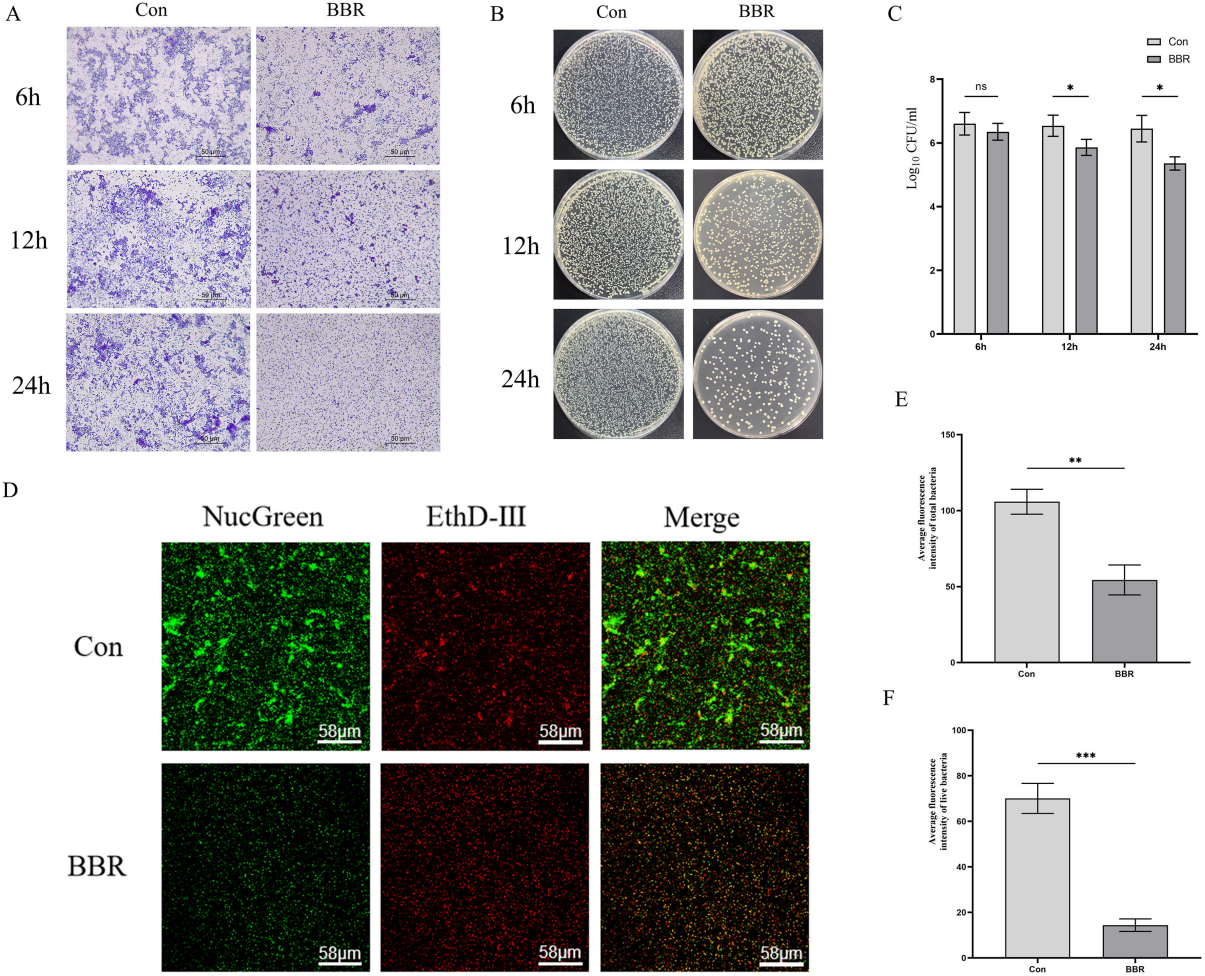
Time-dependent effects of BBR on biofilm structure and viable bacteria within biofilms. **(A)** Crystal violet-stained biofilms after 6, 12, and 24 h of BBR treatment. Scale bar: 50 μm. **(B)** Plate-culture results for bacteria recovered from biofilms. **(C)** Quantitative analysis of colony-forming units (CFU). **(D)** Confocal laser scanning microscopy images showing biofilm architecture and bacterial distribution. Scale bar = 58 μm. **(E)** Mean fluorescence intensity associated with total bacteria within biofilms. **(F)** Mean fluorescence intensity associated with viable bacteria within biofilms. Statistical significance was set at P < 0.05. Symbols denote the observed P-value ranges: *P < 0.05, **P < 0.01, and ***P < 0.001; ns, not significant.

After treatment with BBR for 6, 12, and 24 h, changes in SA biofilm surface coverage were evaluated by crystal violet staining. Biofilm coverage in the control group did not change markedly over time, whereas the crystal violet-positive area in the BBR-treated group progressively decreased with longer treatment. Quantitative analysis showed that, compared with the corresponding control group, the biofilm area in the BBR-treated group decreased by approximately 43.0%, 51.4%, and 68.6% after 6, 12, and 24 h of treatment, respectively, with the greatest reduction observed at 24 h (**Figure 3A**). These findings indicate a time-dependent biofilm-disrupting effect of BBR.

Viable bacteria within the biofilms were further quantified by plate counting. At 6 h, the viable bacterial count in the BBR-treated group showed a downward trend compared with the control group, but the difference was not statistically significant. At 12 and 24 h, viable bacterial counts were significantly lower in the BBR-treated group than in the corresponding control group (**Figures 3B, C**), indicating that prolonged BBR treatment inhibited bacteria embedded within SA biofilms.

### 3.3 Effects of BBR on biofilm structure and bacterial survival at 24 h

Based on these results, the 24-h time point, at which biofilm changes were greatest, was selected for further analysis. CLSM showed that BBR treatment markedly disrupted SA biofilm architecture and significantly reduced both total bacterial fluorescence and live-bacteria-associated fluorescence (**Figures 3D–F**). These findings further support the ability of BBR to reduce bacterial survival within SA biofilms and disrupt biofilm architecture.

### 3.4 Establishment and evaluation of the SA-induced OM mouse model

**Figure 4:**
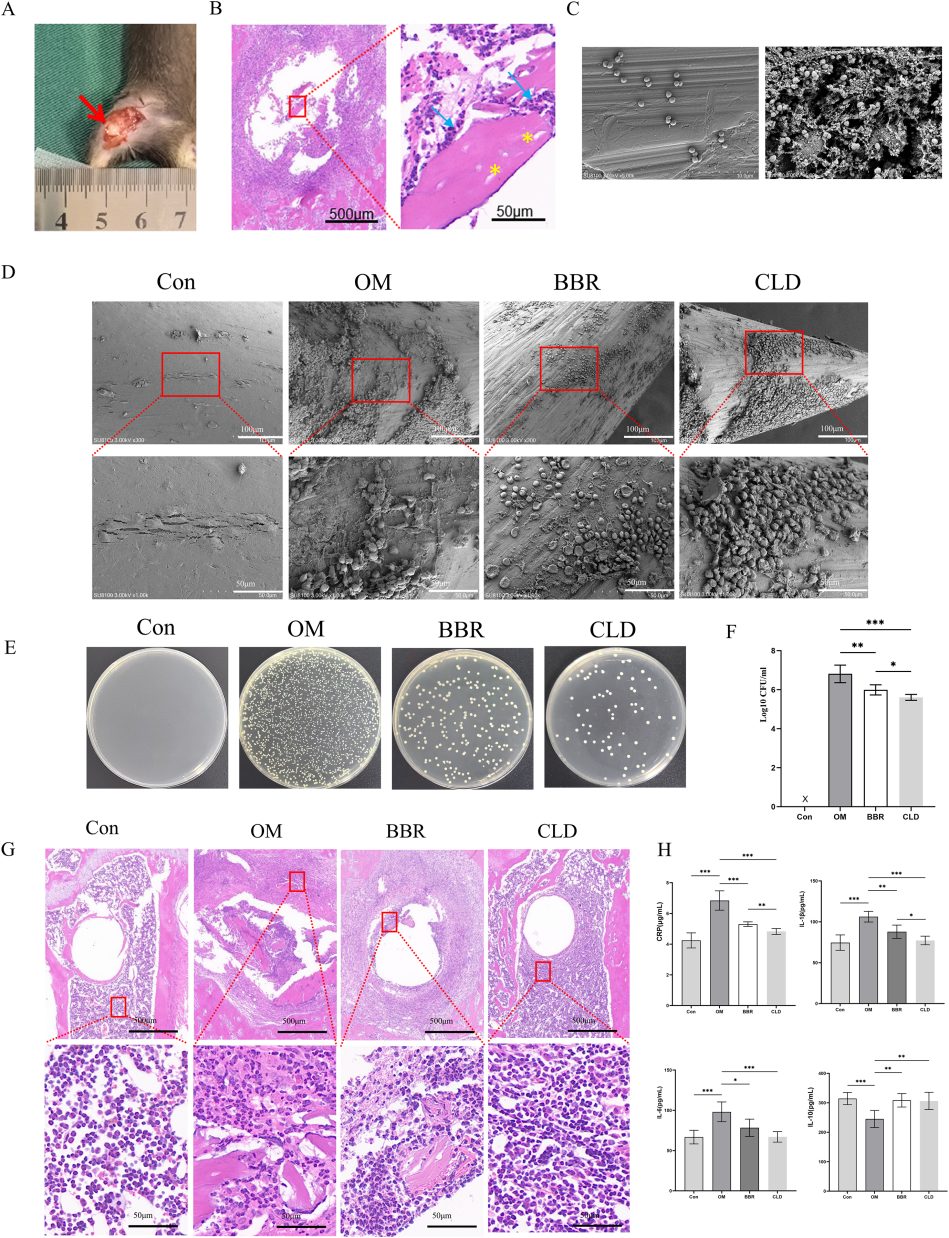
Establishment of a mouse osteomyelitis model and the *in vivo* antibiofilm and anti-inflammatory effects of BBR and CLD. **(A)** Gross appearance of the local tibial region at 3 weeks postoperatively. **(B)** Representative hematoxylin and eosin (H&E)-stained sections of peri-implant tissue. The red-boxed region in the low-magnification image (left) is shown at higher magnification (right). Blue arrows indicate prominent cellular infiltration adjacent to the implant site, whereas yellow asterisks indicate necrotic bone characterized by eosinophilic bone matrix and empty osteocyte lacunae. Scale bars: 500 μm in the original image and 50 μm in the magnified view. **(C)** SEM images showing bacterial colonization and biofilm formation on implant surfaces. The left image shows bacterial adhesion to SA-coated steel pins before implantation, and the right image shows biofilm formation on infected implants retrieved at 3 weeks postoperatively. Scale bar: 10 μm. **(D)** SEM images of implant-surface biofilms in the Con, OM, BBR, and CLD groups. Scale bars: 100 μm in the original images and 50 μm in the magnified views. **(E)** Plate-culture results for bacteria recovered from implant surfaces. **(F)** Quantitative analysis of colony counts. **(G)** H&E-stained bone sections from each group. Scale bars: 500 μm in the original images and 50 μm in the magnified views. **(H)** ELISA results for serum inflammatory mediators (CRP, IL-6, IL-1β, and IL-10). Statistical significance was set at P < 0.05. Symbols denote the observed P-value ranges: *P < 0.05, **P < 0.01, and ***P < 0.001; ns, not significant.

Three weeks after surgery, gross examination revealed obvious subcutaneous abscess formation around the infected steel-pin implantation site in the proximal tibia (**Figure 4A**). H&E staining showed extensive inflammatory cell infiltration and necrotic bone around the implant (**Figure 4B**). SEM confirmed bacterial adhesion to SA-coated implants before surgery. At 3 weeks postoperatively, the retrieved steel pins were covered by dense biofilm-like structures and bacterial aggregates (**Figure 4C**). Together, these findings indicated successful establishment of the SA-induced OM model.

### 3.5 Effects of BBR and CLD on implant-surface biofilms and viable bacteria within biofilms

SEM showed no obvious biofilm formation or bacterial colonization on implant surfaces in the control group (Con). In the OM group, dense three-dimensional biofilm structures with abundant bacterial aggregates were observed on the implant surface. In contrast, biofilm structures in the BBR-treated group appeared less compact, with visibly reduced surface coverage and bacterial burden. Compared with the BBR group, the CLD-treated group showed a more pronounced reduction in biofilm coverage and bacterial colonization (**Figure 4D**).

Plate-counting results further showed that, compared with the OM group, viable bacterial counts recovered from implant-surface biofilms were significantly lower in both the BBR and CLD groups. Moreover, viable bacterial counts were significantly lower in the CLD group than in the BBR group, indicating that CLD exerted a stronger antibacterial effect against implant-associated SA biofilms (**Figures 4E, F**). Nevertheless, BBR also significantly reduced bacterial burden compared with untreated OM mice, supporting its inhibitory activity against implant-associated SA biofilms *in vivo*.

### 3.6 Effects of BBR and CLD on inflammatory responses in OM

H&E staining showed extensive inflammatory cell infiltration and necrotic changes in peri-implant bone tissue in the OM group. After BBR treatment, inflammatory infiltration was markedly alleviated, with fewer inflammatory cells and improved local tissue morphology. Similar histological improvement was observed in the CLD-treated group, with reduced inflammatory cell infiltration and less severe necrotic changes than in the OM group (**Figure 4G**).

ELISA results showed that, compared with the OM group, serum CRP, IL-6, and IL-1β levels were significantly lower in the BBR group, whereas IL-10 levels were significantly higher. CLD treatment also reduced pro-inflammatory mediator levels and increased IL-10 levels relative to the OM group (**Figure 4H**). No statistically significant differences in IL-6 or IL-10 levels were detected between the BBR and CLD groups. These findings indicate that BBR modulated systemic inflammatory responses in SA-induced OM despite its weaker antibacterial effect than CLD; however, the absence of a statistically significant difference should not be interpreted as evidence of equivalence.

### 3.7 Screening of core targets and enrichment analysis

**Figure 5:**
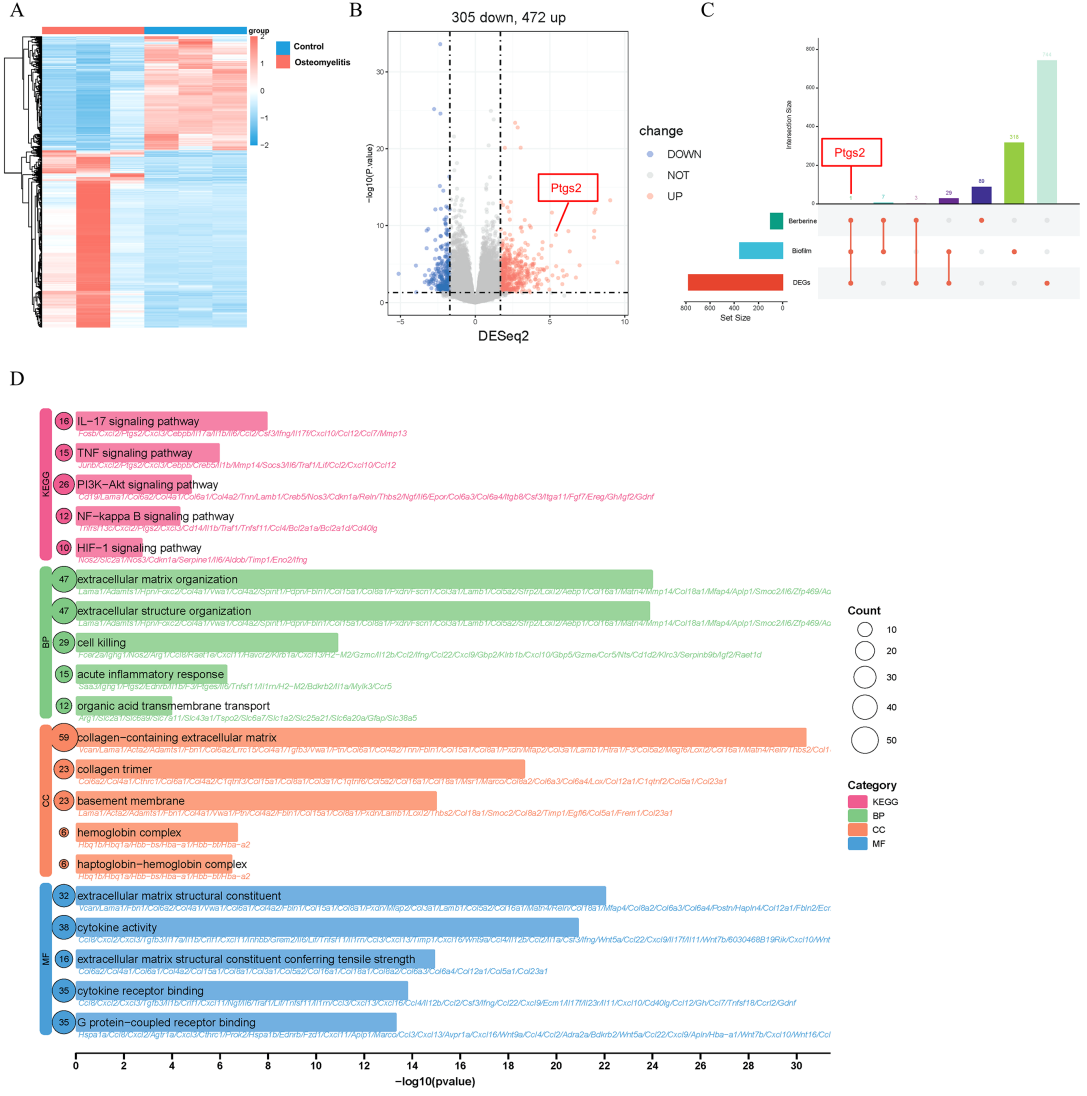
Candidate-target screening and functional enrichment analysis. **(A)** Heatmap of differentially expressed genes (DEGs). **(B)** Volcano plot showing the distribution of DEGs. **(C)** UpSet intersection analysis of predicted BBR targets, SA biofilm-related genes, and OM-associated DEGs. **(D)** KEGG pathway and GO enrichment analyses of DEGs, including biological process, cellular component, and molecular function categories.

The analysis included three OM samples and three matched control samples from GSE166522. Differential expression analysis identified 777 DEGs, including 472 upregulated and 305 downregulated genes (**Figures 5A, B**). SwissTargetPrediction yielded 100 potential BBR targets, and GeneCards yielded 355 SA biofilm-related genes. Intersection of the three gene sets identified PTGS2 as the sole shared target (**Figure 5C**). KEGG enrichment analysis of the DEGs highlighted inflammation-related pathways, including hypoxia-inducible factor 1 (HIF-1), interleukin-17 (IL-17), tumor necrosis factor (TNF), phosphoinositide 3-kinase/protein kinase B (PI3K/Akt), and nuclear factor kappa B (NF-κB) signaling. GO enrichment analysis of the DEGs identified biological processes such as acute inflammatory response, extracellular matrix organization, and cell killing, as well as molecular functions including cytokine activity and receptor binding (**Figure 5D**). These results suggest that BBR may exert therapeutic effects by modulating inflammatory responses and tissue remodeling.

### 3.8 Molecular docking and expression validation

**Figure 6:**
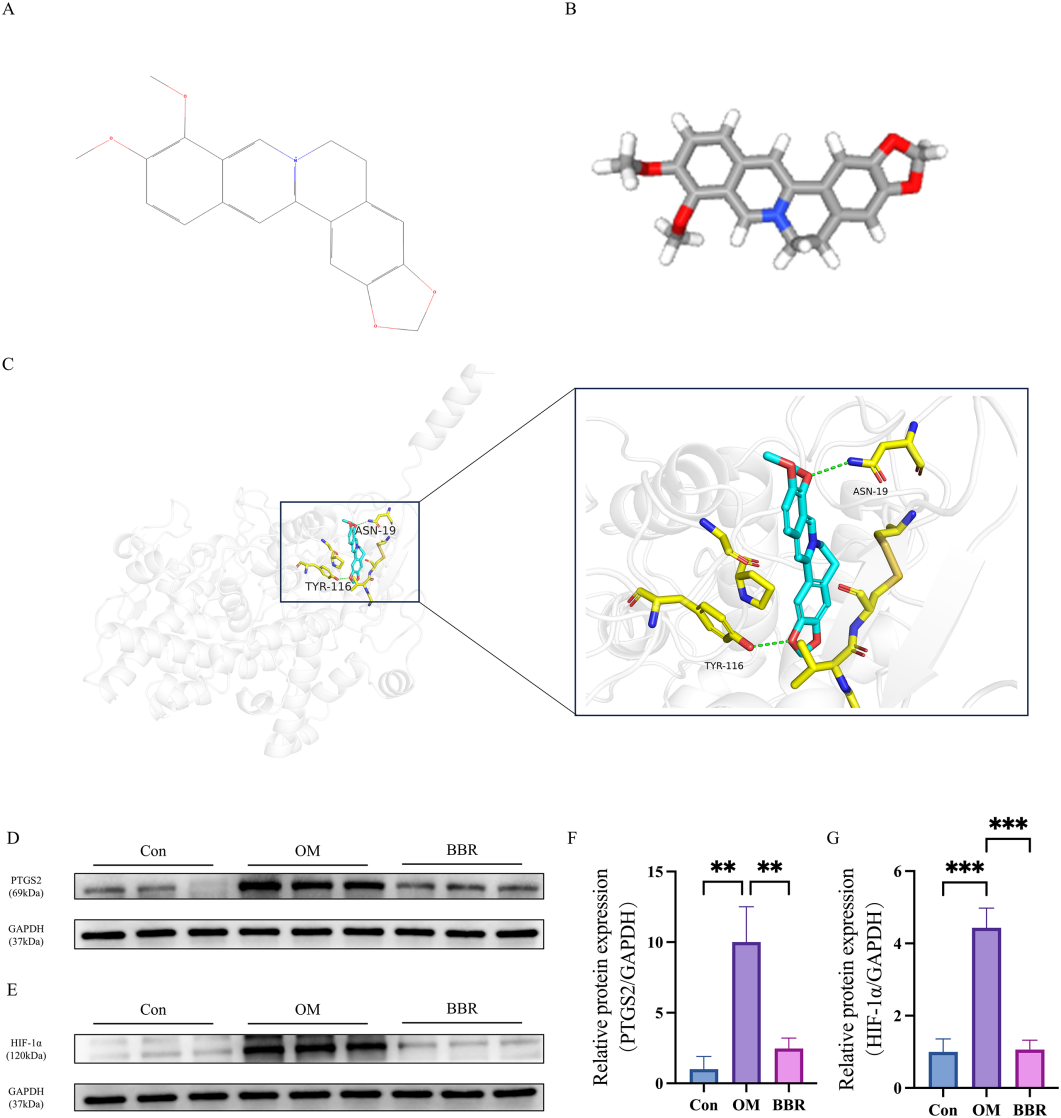
Molecular docking and validation of related protein expression. **(A)** Two-dimensional structure of BBR. **(B)** Three-dimensional structure of BBR. **(C)** Molecular docking mode of BBR with the PTGS2 protein and key binding sites. **(D)** PTGS2 protein expression detected by Western blot. **(E)** HIF-1α protein expression detected by Western blot. **(F)** Grayscale quantitative analysis of PTGS2 protein expression. **(G)** Grayscale quantitative analysis of HIF-1α protein expression. Statistical significance was defined as P < 0.05; *P < 0.05, **P < 0.01, and ***P < 0.001 indicate different P-value ranges after statistical testing, rather than different predefined significance thresholds.

The two-dimensional and three-dimensional structures of BBR are shown in **Figures 6A** and **B**. Molecular docking predicted interactions between BBR and PTGS2 residues Asn19 and Tyr116, with a Vina docking score of −8.3 kcal/mol (**Figure 6C**), suggesting a favorable predicted binding pose. Western blotting showed that PTGS2 and HIF-1α protein levels were significantly higher in the OM group than in the Con group and were markedly reduced after BBR treatment (**Figures 6D–G**). These findings suggest that BBR may modulate PTGS2- and HIF-1α-related inflammatory signaling.

## 4 Discussion

In this study, we systematically evaluated the protective effects of BBR in SA-induced OM using an *in vitro* mature-biofilm model, an *in vivo* mouse model, network pharmacology, and molecular docking. BBR reduced bacterial survival within mature biofilms and disrupted biofilm architecture; it also attenuated inflammatory bone injury and lowered systemic pro-inflammatory mediator levels. Network pharmacology, docking, and protein-expression data further implicated PTGS2- and HIF-1α-related inflammatory signaling. Together, these findings suggest that BBR combines antibiofilm and anti-inflammatory activities in this model of SA-induced OM.

SA is one of the most common pathogens responsible for OM. Its pathogenicity is associated not only with the expression of virulence factors but also with its ability to form biofilms. Biofilms provide a physical barrier, limit antimicrobial penetration, and facilitate bacterial evasion of host immune clearance, thereby promoting persistent infection and recurrence. In implant-associated infection and chronic OM, bacteria frequently persist within biofilms, which contributes to the limited efficacy of conventional antibiotic therapy (24). Therefore, strategies that combine antibiofilm activity with immunomodulatory effects may improve the treatment of OM.

BBR is a natural isoquinoline alkaloid widely found in traditional Chinese medicinal herbs such as *Coptis chinensis* and *Phellodendron chinense*. It has multiple pharmacological activities, including antibacterial, anti-inflammatory, antioxidant, and antitumor effects, and has been investigated in various diseases, particularly those involving metabolic and immune dysregulation (25, 26). However, as a plant-derived compound, BBR has several pharmacological limitations that must be considered when interpreting its therapeutic potential. Its oral bioavailability is very low, mainly because of poor intestinal absorption, P-glycoprotein-mediated efflux, and extensive first-pass metabolism in the intestine and liver (27–29). In the present study, the MIC of BBR against SA was 0.4 mg/mL, whereas the concentration operationally designated as the MBEC against mature biofilms was 3.2 mg/mL, substantially higher than the MIC. This finding is consistent with the increased drug tolerance of bacteria embedded within biofilms compared with planktonic bacteria (30). (1.0) It also indicates that the antibiofilm effect of BBR required relatively high concentrations, highlighting an important limitation of BBR as a plant-derived antimicrobial compound. Nevertheless, BBR significantly reduced viable bacterial counts within biofilms and progressively disrupted biofilm architecture in a time-dependent manner. Crystal violet staining, CFU counting, and confocal microscopy collectively showed reduced biofilm coverage, lower bacterial burden, and decreased live-bacteria-associated fluorescence. These findings indicate that BBR acts against not only planktonic bacteria but also bacteria embedded within mature biofilms.

The mechanisms by which BBR inhibits biofilms are likely multifactorial. Previous studies suggest that BBR can disrupt bacterial cell-wall and membrane integrity, alter extracellular polysaccharide synthesis, suppress adhesion-related factors, and interfere with quorum-sensing systems, thereby inhibiting biofilm formation and maintenance (31). Although the present study did not examine canonical biofilm-associated genes such as *ica*, *sarA*, and *agr*, the observed structural changes and reduction in viable bacterial counts are consistent with effects on bacterial adhesion, aggregation, or biofilm-matrix stability. Transcriptomic, proteomic, and targeted gene-expression studies are needed to define the direct bacterial mechanisms underlying the antibiofilm activity of BBR.

In the *in vivo* experiments, we successfully established an SA-induced OM mouse model. Mice in the model group exhibited key features of implant-associated bone infection, including local abscess formation, dense biofilm coverage on implant surfaces, extensive inflammatory cell infiltration, and bone-tissue destruction. To further assess the limitations of BBR as a plant-derived compound, CLD was included as a positive antibiotic comparator. After BBR treatment, SEM showed reduced biofilm formation on implant surfaces, CFU counting showed a lower bacterial burden, and H&E staining showed less inflammatory infiltration and tissue necrosis. However, CLD reduced implant-associated bacterial burden more effectively than BBR, indicating that the direct antibacterial efficacy of BBR was weaker than that of conventional antibiotic treatment. This result is consistent with the relatively high MIC and operational MBEC values observed *in vitro*. Nevertheless, BBR significantly reduced bacterial load and biofilm formation compared with untreated OM mice, supporting inhibitory activity against implant-associated SA biofilms *in vivo*.

In addition to bacterial persistence, excessive inflammation is an important driver of bone destruction and tissue injury in OM. SA infection activates innate immune responses and promotes the release of pro-inflammatory mediators such as IL-1β, IL-6, and CRP. These mediators can amplify local inflammation, promote bone resorption and tissue necrosis, and thereby aggravate disease progression (5). In this study, BBR treatment significantly reduced serum CRP, IL-6, and IL-1β levels while increasing the anti-inflammatory cytokine IL-10. No statistically significant differences in serum IL-6 or IL-10 levels were detected between the BBR and CLD groups, although this finding does not establish equivalence between the treatments. Together with the H&E results, these data suggest that the protective effect of BBR is not attributable solely to antibacterial activity but also involves modulation of inflammatory responses and attenuation of host-mediated tissue injury. This may be relevant to chronic bone infection, in which bacterial killing alone may not reverse ongoing bone destruction driven by inflammatory cascades (7). Thus, BBR should not be regarded as a replacement for antibiotics but may warrant further investigation as an adjunctive agent with both antibiofilm and immunomodulatory properties.

To explore potential mechanisms, we integrated predicted BBR targets, OM-associated DEGs, and SA biofilm-related genes and identified PTGS2 as the sole shared gene. PTGS2, also known as cyclooxygenase-2 (COX-2), is an inducible enzyme that catalyzes the conversion of arachidonic acid to prostaglandin intermediates and plays an important role in inflammation, tissue injury, and immune responses (32). PTGS2 is upregulated in numerous infectious and inflammatory conditions and can contribute to inflammatory mediator production, vasodilation, pain, and tissue injury (33–35). PTGS2-related signaling also intersects with inflammatory pathways involving HIF-1, NF-κB, TNF, PI3K/Akt, and IL-17 (36–38). In the present study, enrichment analysis of OM-associated DEGs—not of PTGS2 alone—highlighted these inflammation-related pathways. The convergence of PTGS2 with these pathway-level findings provides a mechanistic hypothesis for further testing but does not by itself establish PTGS2 as the causal mediator of BBR.

HIF-1α, the principal oxygen-sensitive transcriptional regulator of the HIF-1 pathway, participates not only in hypoxic adaptation but also in inflammatory amplification, immunometabolic reprogramming, and tissue repair during infection (39). In the bone-infection microenvironment, bacterial proliferation, inflammatory-cell infiltration, and microcirculatory dysfunction can produce relative hypoxia and promote HIF-1α activation (40). HIF-1α can also interact functionally with inflammatory regulators such as NF-κB and PTGS2, thereby influencing cytokine production and tissue injury (41). Together with the enrichment results, these observations suggest that HIF-1-related signaling may be involved in the response to BBR in OM.

Molecular docking yielded a favorable predicted pose of BBR within PTGS2, with a Vina score of −8.3 kcal/mol and predicted contacts with Asn19 and Tyr116. However, docking scores alone do not demonstrate direct binding or functional inhibition. Western blotting showed that PTGS2 and HIF-1α were upregulated in OM lesion tissue and reduced after BBR treatment, consistent with the computational predictions. These data support the hypothesis that BBR may influence PTGS2- and HIF-1α-related inflammatory signaling, but direct target engagement, pathway directionality, and downstream prostaglandin changes require further experimental validation.

Biofilms and inflammatory responses are not independent processes but can form a mutually reinforcing cycle. Biofilms promote persistent bacterial colonization and immune evasion, resulting in chronic inflammatory stimulation. Conversely, cytokines, tissue debris, and necrotic products within the inflammatory microenvironment may facilitate bacterial adhesion and biofilm persistence (42–44). Simultaneously targeting biofilm persistence and excessive inflammation may therefore be advantageous in SA-induced OM. The present findings suggest that BBR affects both components of this cycle, although the causal links between its antibiofilm and immunomodulatory actions remain to be defined.

The translational limitations observed for BBR are not unique among plant-derived antimicrobial compounds. Several phytochemicals, including curcumin, resveratrol, and quercetin, show anti-staphylococcal or antibiofilm activity *in vitro*, but their therapeutic development has been constrained by insufficient potency, poor aqueous solubility, low systemic bioavailability, rapid metabolism, chemical instability, or formulation-related barriers (45). For example, curcumin has antimicrobial activity against pathogens associated with surgical and implant-related infections, including S. aureus, but its poor water solubility, low bioavailability, and rapid degradation limit clinical translation (46, 47). Resveratrol has also been investigated as an antimicrobial phytochemical, but its systemic bioavailability is very low because of rapid intestinal and hepatic metabolism (48). Quercetin can inhibit MRSA biofilm formation by targeting biofilm-associated regulators such as SarA, but its pharmaceutical development is limited by poor bioavailability, aqueous solubility, permeability, and stability (49, 50). BBR has a similar translational profile: it combines multitarget antibacterial and immunomodulatory activities, but its direct antibacterial potency and pharmacokinetic properties remain important barriers. These comparisons support further development of BBR as an adjunctive or formulation-optimized antibiofilm and anti-inflammatory agent rather than as a stand-alone substitute for antibiotics.

Although this study yielded consistent findings across several experimental approaches, it has limitations. First, although CLD was included as a positive antibiotic comparator, potential synergistic or additive effects of BBR combined with conventional antibiotics were not evaluated. Second, the pharmacokinetics, oral bioavailability, and bone-tissue distribution of BBR were not assessed; therefore, it remains unclear whether the concentration used *in vitro* can be achieved in infected bone tissue. Third, the network-pharmacology intersection yielded only one shared target, PTGS2, and may not capture the broader multitarget effects of BBR. Fourth, changes in PTGS2 and HIF-1α expression were validated, but direct target engagement and upstream or downstream relationships involving prostaglandins, NF-κB, mitogen-activated protein kinase (MAPK), and PI3K/Akt signaling were not investigated; thus, the mechanistic interpretation remains preliminary. Fifth, the *in vitro* experiments primarily evaluated biofilm surface coverage, fluorescence signals, and viable bacterial counts; biofilm-matrix components, adhesion-related genes, quorum-sensing signals, and virulence factors were not examined. Sixth, the animal sample size was limited, and only one bacterial strain and one dosing regimen were tested. Future studies should evaluate additional strains, including drug-resistant isolates, dose-response relationships, pharmacokinetics, and combination therapy. Finally, optimized delivery strategies, such as bone-targeted formulations, local delivery systems, nanocarriers, or antibiotic combinations, may be required to overcome the limited bioavailability and antibacterial potency of BBR.

## 5 Conclusion

This study demonstrates that BBR can inhibit SA biofilms, reduce bacterial survival within biofilms, and alleviate OM-associated inflammation and bone-tissue injury. The CLD comparator showed that the direct antibacterial efficacy of BBR was weaker than that of conventional antibiotic treatment. Nevertheless, BBR significantly modulated inflammatory mediators; no statistically significant differences in serum IL-6 or IL-10 were detected between the BBR and CLD groups, although this does not establish equivalence. Network pharmacology, molecular docking, and Western blotting implicated PTGS2- and HIF-1α-related signaling as a potential mechanism underlying the protective effects of BBR. These findings support further investigation of BBR as an adjunctive candidate for SA-induced OM and as a basis for developing natural-product strategies with combined antibiofilm and anti-inflammatory activities.

## Data Availability

The original contributions presented in the study are included in the article/**Supplementary Material**, further inquiries can be directed to the corresponding author.
