# Supplementary figures and images for: Berberine alleviates biofilm-associated immune-inflammatory injury in Staphylococcus aureus-induced osteomyelitis: insights from network pharmacology and experimental validation
Source: Front Immunol. 2026 Jul 13;17:1878634. doi: 10.3389/fimmu.2026.1878634 (PMC13402166; doi:10.3389/fimmu.2026.1878634)

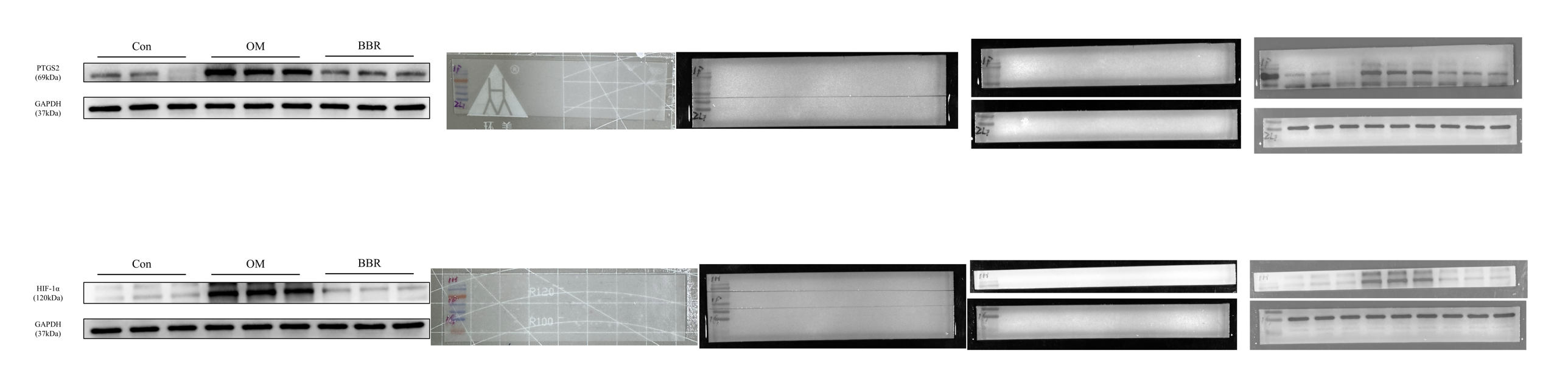

Supplement: Supplementary file 1 [file Image1.tif]
